# Avian influenza at animal‐human interface: One‐health challenge in live poultry retail stalls of Chakwal, Pakistan

**DOI:** 10.1111/irv.12718

**Published:** 2020-02-07

**Authors:** Mamoona Chaudhry, Richard Webby, David Swayne, Hamad Bin Rashid, Jennifer DeBeauchamp, Lindsay Killmaster, Miria Ferreira Criado, Dong‐Hun Lee, Ashley Webb, Shumaila Yousaf, Muhammad Asif, Qurat ul Ain, Mirwaise Khan, Muhammad Ilyas Khan, Saima Hasan, Arfat Yousaf, Abida Mushtaque, Syeda Fakhra Bokhari, Muhammad Sajid Hasni

**Affiliations:** ^1^ Disease Surveillance Laboratory Department of Epidemiology and Public Health University of Veterinary and Animal Sciences Lahore Pakistan; ^2^ Department of Infectious Diseases World Health Organization Collaborating Center for Studies on the Ecology of Influenza in Animals and Birds St. Jude Children's Research Hospital Memphis TN USA; ^3^ Exotic and Emerging Avian Viral Diseases Research Unit Southeast Poultry Research Laboratory U.S. National Poultry Research Center U.S. Department of Agriculture, Agricultural Research Service Athens GA USA; ^4^ Department of Clinical Medicine and Surgery University of Veterinary and Animal Sciences Lahore Pakistan; ^5^ Department of Pathobiology and Veterinary Science University of Connecticut Storrs CT USA

**Keywords:** avian influenza, avian influenza viruses, birds, H9N2, live bird market butchers, Pakistan, risk factors, seroprevalence, surveillance

## Abstract

**Background:**

Live poultry retail stalls (LPRSs) are believed to be the source of human infection with avian influenza viruses (AIVs); however, little is known about epidemiology of these viruses in LPRSs of Pakistan**.**

**Objectives:**

The current study was conducted to estimate the virological and serological prevalence of AIVs in humans and poultry and associated risk factors among seropositive butchers.

**Methods:**

A field survey of LPRSs of Chakwal District was conducted between December 2015 and March 2016. In total, 322 samples (sera = 161 and throat swab = 161) from butchers and 130 pooled oropharyngeal swabs and 100 sera from birds were collected. Baseline sera (n = 100) from general population were also tested. Data were collected by structured questionnaires. Sera were tested by hemagglutination inhibition (HI) test further confirmed by micro‐neutralization test (MN). Swabs were processed by real‐time RT‐PCR. Logistic regression analyses were conducted to identify risk factors**.**

**Results:**

In butchers, 15.5% sera were positive for antibodies against H9 virus using a cutoff of ≥40 in HI titer; 6% sera from general population were positive for H9. Seroprevalence in poultry was 89%, and only 2.30% swabs were positive for H9. Presence of another LPRS nearby and the number of cages in the stall were risk factors (OR > 1) for H9 seroprevalence in butchers.

**Conclusions:**

This study provides evidence of co‐circulation of H9 virus in poultry and exposure of butchers in the LPRSs, which poses a continued threat to public health. We suggest regular surveillance of AIVs in occupationally exposed butchers and birds in LPRSs.

## 1 INTRODUCTION

About hundred years ago, the 1918 influenza pandemic devastated entire communities and was the most severe pandemic in recent history, sweeping the globe quickly and killing more than 50 million people.^1^ The emergence of novel influenza strains through mutation and reassortment from avian reservoir remains a constant threat to animal and public health,^2^ which has been illustrated by multiple human cases due to novel H7N9 in China.^3^ Phylogenetic analysis of these H7N9 viruses indicated that different gene segments were closely related to different AIV strains isolated from domestic ducks and wild birds in South‐East Asia.^4^ In addition, sporadic human infections with symptoms ranging from mild conjunctivitis and influenza‐like illness (ILI) to pneumonia and multi‐organ failure caused by multiple AIVs subtypes have been reported globally.^4^, ^5^, ^6^, ^7^, ^8^ Most of these reports recognized recent exposure to poultry as the most probable cause of infection.^9^, ^10^

Multidisciplinary teams have dealt with emerging infectious diseases, for example, SARS, H5N1 AIV, and the 2009 H1N1 pandemic^11^ by adopting One‐Health strategy. The exponential growth of the human population has elevated the importance of human‐animal‐environment interface.^12^ In addition, traditional small‐scale poultry production systems have been transformed into industrial integrated operations in most parts of the world including Asia.^13^, ^14^ Various studies have been conducted targeting the animal‐human interface and have reported different routes of human exposure to AIVs identifying potential host determinants that favored persistence of virus.^4^, ^5^, ^6^, ^7^, ^8^, ^9^, ^15^, ^16^, ^17^

Early preparedness for global pandemics has become an important public health task. Preparedness includes identification of determinants of disease spread at animal‐human interface and ultimately recognizing areas to implement disease prevention and outbreak control.^14^ Surveillance of domestic poultry and poultry handlers has provided evidence that AIVs are evolving continuously^10^, ^18^, ^19^ and are able to cross species barrier and infect mammals.^7^, ^20^, ^21^, ^22^, ^23^, ^24^ Plentiful evidence is, thus, available to support the phenomenon of interspecies transmission of AIVs globally; nevertheless, very little information is available about the situation in Pakistan.^8^, ^15^, ^18^, ^25^, ^26^ The main objective of the current study was to understand the role of LPRSs in poultry‐to‐human transmission of AIVs. We tried to eliminate bias in estimates by conducting survey and analysis with simple random sampling. We also linked human data with poultry data to identify any association among them. Very few epidemiological surveys based on probability sampling methods have been conducted in developing countries due to lack of baseline data, and this study could provide appropriate design and information for other research to plan a survey with limited resources and data. We found H9N2 virus infection in birds in the LPRSs and evidence for their spillover to occupationally exposed humans.

### 1.1 Ethics committee approvals

Ethical Review Committee for Animals (DR:20) and Independent Ethical Committee for Human, Bioequivalence Center of University of Veterinary and Animal Sciences (UVAS), Lahore, Pakistan approved the research protocol. Permission to conduct survey in LPRS was also obtained from the Commissioner Office of the District Chakwal. Before enrollment, written informed consent from the workers was obtained. The team members who collected samples from human and birds were registered with the respective medical and veterinary accreditation bodies (Pakistan Medical and Dental Council and Pakistan Veterinary Medical Council). They were trained to collect data and conduct interview at UVAS and were also given training about biosafety through a workshop.

## 2 MATERIALS AND METHODS

### 2.1 Study design

**Figure 1:**
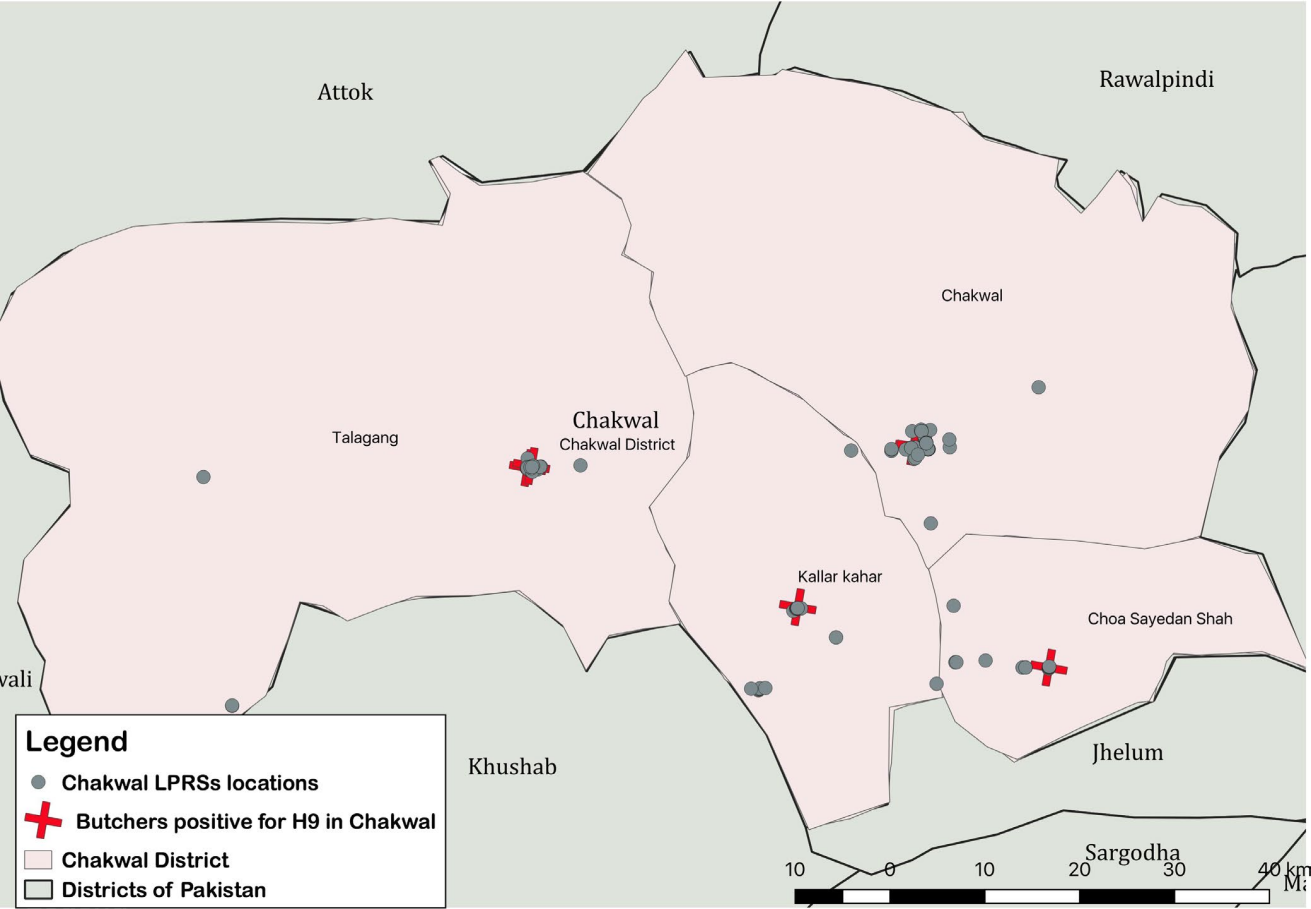
Sampling District (Chakwal) with locations of LPRSs selected for survey and locations of shops with seropositive butcher with H9

The study was conducted in District Chakwal which falls at latitude 32º 56’ N and longitude 72º 53‘ E, with an area of 6,609 square kilometers (http://www.citypopulation.de/Pakistan-100T.html). Chakwal consists of four subdivisions, namely Chakwal, Talagang, Choa Saidan Shah, and Kallarkahar (https://www.punjab.gov.pk/chakwal) (Figure 1). The human population of Chakwal is 1 495 982 (Pakistan Bureau of Statistics, 2017), and there are approximately 4 990 080 commercial broiler birds on approximately 1800 poultry farms.^27^ The control subjects (n = 100) were also selected from Chakwal District.

A cross‐sectional survey of LPRSs in four subdivisions of Chakwal (Figure 1) was conducted from December 2015 to March 2016 to determine the prevalence of circulating AIVs in butchers and in chickens being sold in those LPRSs and to identify the potential risk factors associated with estimated prevalence. According to the Commissioner Office of Chakwal, the total number of functional LPRSs in Chakwal was 276. Live poultry sold in these markets included mostly chickens (layer, broiler, and indigenous/desi), and rarely ducks, geese, pigeons, and quail also. However, samples were collected only from broiler birds in the stalls. The sample size calculations were performed using epiR package in R software.^28^ A simple random method was used to calculate the desired sample size.^29^ The estimated sample size was 161 assuming 50% seroprevalence,^15^, ^30^ 95% confidence, and absolute precision of ±5% with population size of 276 for Chakwal District. The estimated (a priori) prevalence of 50% was used to get the largest sample size to increase the desired precision of estimate.^29^ A list of randomly selected butchers (n = 161) was drawn by random digit generator (stattrek.com/statistics/random‐number‐generator.aspx) from the LPRSs list (N = 276). Our field teams (Veterinarian and Paramedical staff) visited the 161 selected LPRSs for consent and enrollment of butchers (Figure 1).

In addition, serum samples from the general community that were available in the repository of a private laboratory of Chakwal District were included as control subjects. The sera from control subjects having no exposure to poultry or livestock during their daily routine were collected by the registered phlebotomist at the laboratory, who was referred by medical personnel for routine screening for medical diagnosis. The laboratory labeled these samples anonymously to keep the confidentiality of patients. Only data about sex, age, education, and pre‐existing history of chronic disease were available in records.

From each LPRS, one worker was enrolled after taking consent. Inclusion criteria for butchers included ≤15 years of age, having worked in LPRSs for more than six months, having exposure to poultry slaughtering as part of daily activities for more than 8 h/wk (slaughtering and sales). Exclusion criteria included having severe respiratory illness in last three months, known history (self‐reporting) of human immunodeficiency virus (HIV), tuberculosis, and those who refused to participate at the time of enrollment. No participants received a human seasonal influenza vaccine. A standard questionnaire was used to collect epidemiological information, including demographic characteristics, occupational exposure (slaughtering, selling, and handling poultry), and other potential risk factors of exposure (Appendix S1). Risk factors included education of respondent, age of respondent, smoking, having any chronic disease, stall remained open, number of cages, selling birds other than broiler, adding newly arrived birds to cages already having birds, presence of wild bird, presence of rodents, access of stray dogs, access of stray cats, preparing raw poultry using knife, touching face/food, washing facility in market, washing instruments, washing hands, wearing mask, wearing gloves, wearing protective boots, wearing apron, covering nose and mouth with handkerchief, and put carcass on ground/in drum, clean cutting board, washing gizzard, wash stall daily, another stall nearby. A trained team member administered the questionnaire in a face‐to‐face interview with the participant.

Each enrolled participant allowed serum and throat swab collection. From the general population, sera were collected from any age group. Information about sex, age, education, and pre‐existing chronic illness history of general community participants was available in laboratory repository records. Approximately 3‐5 mL of blood samples was collected by venipuncture, and serum was separated and stored at −20°C until serological analysis (DSL, UVAS, Lahore and WHO Collaborating Center for Avian Influenza Viruses, St. Jude Children's Research Hospital, Memphis, Tennessee, USA). Throat or nasopharyngeal swab specimens obtained from participants were maintained in a viral transport medium (Brain heart infusion broth with antibiotics, Oxoid no. CM1135, Oxoid, UK) and were transported on ice to the DSL, UVAS, Lahore, where they were coded and stored at −80°C until laboratory analysis.^31^

Butchers were also requested to provide oropharyngeal swabs, and blood samples from the broiler birds present in the stall at the time of survey. Apparently, healthy chickens were selected from the LPRSs after taking informed consent of stall owner. Selection of birds was an arbitrary choice of the butcher. All birds included in the study were adult broiler birds commercially available for slaughter. Only 130 stall owners allowed oropharyngeal swab to be taken from their birds, and 100 stall owners allowed collection of blood from their birds during slaughtering. A trained veterinarian collected pooled oropharyngeal swabs, that is, at each LPRS individual swabs from 5 birds. Swabs were collected and pooled in one tube containing viral transport medium. Blood was collected from one bird at each stall.

### 2.2 Laboratory analysis

The hemagglutination inhibition (HI) test was used to screen human sera^31^ and poultry sera.^32^ Sera from butchers were tested in duplicate by parallel testing in US and Pakistan, and each assay included specific positive (chicken hyperimmune antiserum against specific subtype antigens) and negative control (sera from non‐exposed adults) sera. Briefly, sera were treated with receptor destroying enzyme (RDE; Denka Seiken Co. Ltd to eliminate non‐specific inhibitors. One volume of serum (50 μL) was added to 3 volume of reconstituted RDE (150  μL) and incubated at 37**°**C overnight. After that sera were heat inactivated at 56**°**C for 30 minutes. Finally, 6 volume of phosphate buffered saline (PBS) (300  μL) was added to make final dilution of 1:10 (500 ul total volume of prepared sera). Turkey RBCs (0.5%) were used to perform HI tests in U‐bottom 96‐well micro‐titration plates. At DSL, UVAS, chicken RBCs (0.5%), and V‐bottom 96‐well titration plates were used for HI test. The HI test results were reported at antibody cutoff titers ≥40, ≥80, and ≥160. For this study, HI antibody titer ≥1:40 was considered positive for H9, H5, and H7. This cutoff has been used previously to record previous infection.^17^, ^24^, ^33^, ^34^ All sera of butchers having antibody titer of ≥1:20 against H9 in HI test were further tested by micro‐neutralization (MN) assay for the detection of antibodies with H9 avian influenza candidate vaccine virus RG‐A/Bangladesh/0994/2011‐A/PR/8/34 [R] (6 + 2). All MN assays were performed with MDCK cells according to the WHO manual, with an MN cutoff value of 1:20.^31^ The MN assay was conducted at WHO Collaborating Center for Avian Influenza Viruses, St. Jude Children's Research Hospital, Memphis, Tennessee, USA.

We also conducted HI of 100 sera from poultry collected from retail shops and tested against H9, H7, and H5 with a cutoff titer ≥16 as described in OIE manual.^32^

For HI test of human sera, antigens derived from A/Quail/Bangladesh/19462/2013 (H9N2), A/duck/Bangladesh/19097/2013 (H5N1), and A/Netherlands/219/2003 (H7N7) viruses were used. For the latter two viruses, antigens were derived from attenuated versions of the virus that were devoid of the polybasic amino acid connecting peptides associated with high virulence.

Viruses were propagated in the allantoic cavities of 10‐day‐old embryonating hen eggs. After 48 hours post‐inoculation, allantoic fluid was harvested. Hemagglutination (HA) assay was used to determine the titer of virus using 0.5% chicken red blood cells (RBCs). Confirmation of the reference viruses was done by using monoclonal sera raised against each virus.^31^

Real‐time reverse transcription PCR (rRT‐PCR)^35^ described by Centers for Disease Control and Prevention (CDC; Atlanta, GA, USA) was used to initially screen human swabs for influenza A virus (IAV). The cutoff cycle threshold (Ct) value for influenza positive swab specimen was ≤37^6^; samples having Ct values between 37 and <40 were designated as suspected; and samples with >40 Ct values were considered negative. The rRT‐PCR for human swab was conducted at World Health Organization Collaborating Center for Studies on the Ecology of Influenza in Animals and Birds, Department of Infectious Diseases, St. Jude Children's Research Hospital, Memphis, TN, USA.

The oropharyngeal swabs from chickens in LPRSs were tested for AIV matrix gene at Exotic and Emerging Avian Viral Diseases Research Unit, Southeast Poultry Research Laboratory, US National Poultry Research Center, Agricultural Research Service, US Department of Agriculture, Athens, GA, USA. Briefly, RNA extraction of virus was done by using MagMAX™‐96 AI/ND Viral RNA Isolation Kit^®^ (Thermo Fisher Scientific) according to the manufacturer's instruction. After the extraction, the rRT‐PCR analysis for matrix gene was conducted as published^36^ with modification.^37^ Samples were run and analyzed on the 7500 FAST real‐time PCR System (Applied Biosystems). Positive samples for AIV were further subtyped for H5, H7,^38^ and H9^39^ at UVAS. The cutoff Ct value for influenza positive swab specimen was <34.

### 2.3 Data analysis

R software was used to conduct all statistical analyses.^28^ Point estimates with 95% confidence intervals (CI) were calculated in survey package for R software.^40^ Two‐sample test of proportions (Z test) was performed to test the equality of proportions for seroprevalence among butchers, poultry and general population, virological prevalence between butchers and poultry birds, and seroprevalence in butchers at different cutoff values (1:40, 1:80, 1:160, and ≥1:40), gender, age categories, pre‐existing chronic medical conditions, contact with poultry. Unconditional logistic regression was used to conduct multivariable analysis for binary HI outcomes for butcher data set. Variables with *P* < .25 were selected for multivariable analysis adopting the manual forward elimination using *P* < .05 to select the final model. The location of each LPRSs was recorded with a smart phone app Google map. Maps were created in QGIS version 3.2.2 Bonn (available at http://qgis.org/). Shape files of Pakistan boundaries, administrative division, etc were downloaded from the internet (http://www.diva-gis.org/datadown and http://www.mapcruzin.com/free-pakistan-arcgis-maps-shapefiles.htm). Using available geographical data, dot map of spatial distribution of LPRS in Chakwal District was generated.

## 3 RESULTS

Among enrolled participants (butchers = 161; control subjects = 100), majority were in the age group 15‐30 years (56.43%). The median age of butchers was 28 (range 15‐65), and all were male (100%). The median age of control subjects was 27 years (range 0‐70), and 33% were male while 67% were female (Table 1). None of the participants reported receiving seasonal influenza vaccine or monovalent H1N1 2009 vaccine.

**Table 1:** Demographic characteristic of study participants

Characteristics of population	Poultry butchers (n = 161)	Control subjects (n = 100)	* *P *value
Sex, participants			
Male	161 (100)	33 (33)	<.001
Female	0 (0%)	67 (67)	‐
Age, years, no. (%)			
0‐14	0 (0%)	7 (7)	‐
15‐30	96 (59.60)	51 (51)	.171
31‐45	51 (31.7)	35 (35)	.578
46‐60	13 (8.1)	4 (4)	.194
61‐above	1 (0.6)	3 (3%)	.128
Education, no. (%)			
Uneducated	51 (31.7)	29 (29)	.64
Primary	56 (34.8)	21 (21)	.017
Secondary and above	54 (33.5)	50 (50)	.008
Contact with poultry	161 (100)	‐	‐
Medical history of chronic disease conditions (Liver, Kidney, Heart disease, etc	45 (27.95)	30 (30)	.722

* *P* value calculated for two‐sample test for equality of proportion

Most of LPRSs visited, conducted business 7 days a week (97.5%) with only 2.5% opening for 6 days. In terms of store capacity, 50.9% of stalls kept one cage (range 1‐9) with a capacity of 50 birds (26.1% stalls) and average turnover of 64 birds sold per day (range 20‐250). About 58.4% (n = 94) LPRSs also kept indigenous breeds of poultry along with commercial poultry.

### 3.1 Seroprevalence of AIV H9 antibody in butchers, general community, and poultry

None of the sera from butchers and control subjects were positive for antibodies against H7 and H5. Overall, 15.5% (95% CI%: 12.1‐20.0) of butchers (25/161) were positive for H9 antibodies using an HI titer cutoff of ≥40 (Table 2, Figure 1). Thirty‐nine (39) samples with HI titer of ≥20 were tested by MN test. Overall, 31 subjects (31/39 = 79.48%) were positive by either HI or MN assays using cutoff antibody titer of ≥40 for HI and ≥20 for MN. Fifteen subjects (15/39 = 38.46%) were common positives in both HI and MN assays. Eight samples were common negative, 10 samples were positive by HI and negative by MN test, and 6 samples were positive by MN but negative by HI (Table 3). The likelihood of a sample being tested positive was 4.75 times more when using a HI cutoff of ≥40 as compared to ≥80 (*P* < .001) and likelihood of a sample being tested positive was 1.53 times more when using a HI with cutoff of ≥40 as compared to MN at cutoff of ≥20 (*P* > .05). Our results show that the seropositivity against H9 at a cutoff of ≥40 was much higher than that at cutoff ≥80 (15.5% vs 3.72%, *P* < .001). The seropositivity with HI test against H9 in butcher was also significantly higher than general community (15.5% vs 6.00%, *P* < .05). Among the H9 seropositive samples, 51% belonged to the 15‐30 years of age group, while 35% were from the 31‐45 years of age group. Only 4% were aged 46‐60 years. There was no significant association between age category and seropositivity against H9 (*P* > .05). All poultry sera collected from LPRSs were negative for H5 and H7. The seroprevalence of H9 in poultry was 89% (95% CI%: 81.16‐94.37), and it was significantly associated with seroprevalence of H9 in butchers (89.00% vs 15.5%, *P* < .001).

**Table 2:** Distribution of antibodies titers against AIV H9 in Hemagglutination Inhibition test in butchers and general population

HI dilution for H9	No. (%)	
	HI test results	HI test results
	Butchers (n = 161)	General population (n = 100)
<10	117 (72.7)	73 (73%)
1:10	5 (3.1)	17 (17%)
1:20	14 (8.7)	4 (4%)
1:40	18 (11.2)	5 (5%)
1:80	6 (3.7)	1 (1%)
≤160	1 (0.6)	0
≥1:40	25 [15.5%, (12.1‐20.0)]	6 [6%, (2.23‐12.60)]

**Table 3:** Distribution of antibodies titers against AIV H9 in Hemagglutination Inhibition test and Micro‐neutralization Test in butchers’ sera

HI dilution for H9	MN dilutions for H9						
	N/A	<10	1:10	1:20	1:40	1:80	Total
<10	117	0	0	0	0	0	117
1:10	5	0	0	0	0	0	5
1:20	0	4	4	4	2	0	14
1:40	0	5	2	4	6	1	18
1:80	0	1	1	0	2	2	6
≤160	0	0	1	0	0	0	1
Total	122	10	8	8	10	3	161

### 3.2 Prevalence of AIV in butchers and birds in LPRSs

Among the 161 throat swabs examined, none were positive for matrix gene of influenza A (Ct > 37). Seven samples (3.1%) showed insufficient virus quantity (Ct 37‐40) by rRT‐PCR test.

Out of 130 pooled oropharyngeal swabs from 650 birds, 32 samples were suspected positive for AIV M gene having Ct values ≤ 40. Of these, 32 suspected samples, 11 (8.46%) with Ct values < 34, were further processed for subtyping by rRT‐PCR assay (H5 & H7) and conventional RT‐PCR (H9). Out of 11 samples, 3 (2.30%) were positive for H9 through conventional RT‐PCR. We confirmed by rRT‐PCR that all M gene‐positive samples were negative for H5 and H7 genotype. Significant association (*P* < .001) was found in H9 seroprevalence in butchers (25/161, 15.5%) and H9 virus prevalence in poultry birds in the same stalls (3/130, 2.30%). Similarly, seroprevalence of H9 in birds in LPRSs was also significantly associated with prevalence of H9 virus in same retails stalls (89% vs 2.3%, *P* < .001).

### 3.3 Risk factors for AIV H9 seroprevalence in butchers

Thirty‐three variables were screened in a univariable analysis, and 6 variables showed association with seroprevalence of H9 in butchers (Table 4). In multivariable analysis, two variables namely “another stall nearby” and “number of cages” remained strongly associated with seroprevalence of H9 (*P* < .05) (Table 5).

**Table 4:** Risk factors in univariable analysis associated with seroprevalence of H9 in butchers

Variables	H9‐positive subjects	H9‐negative Subjects	*P* value
Number of cages			
Less than 5	22	131	.012
More than 5	3	5	
Another stall nearby			
No	6	66	<.001
Yes	19	70	
Washing facility in market			
No	21	124	.095
Yes	4	12	
Washing gizzard			
Separately under tap	3	9	.153
Dip in bucket of water	22	127	
Clean cutting board			
No	24	121	.113
Yes	1	19	
Wash stall daily			
No	21	124	.095
Yes	4	12	

**Table 5:** Results of multivariable unconditional logistic regression analysis

Risk factor	Response	Odds ratio	95% Confidence Interval (CI)	*P* value
Another stall nearby	No	1		<.001
	Yes	3.38^a^	1.78‐6.39	
Number of cages	Less than 5	1		<.05
	More than 5	4.90^b^	1.60‐14.97	

a The butcher in a stall having another LPRS nearby was 3.38 (CI 95%: 1.09‐19.3) times more likely to become seropositive with H9 when compared to a butcher in a stall having no other stall nearby.

b The odds of having more than 5 cages in a stall for seropositive butchers were 4.90 (95% CI: 1.60‐14.97) times greater than the odds of exposure in the seronegative butchers.

## 4 DISCUSSION

Our study results suggest subclinical infection of butchers with H9 as these workers self‐reported no history of severe respiratory illness on enrollment. Absence of exposure to H5 and H7 AIVs was also documented as none of the sera from butchers and control subjects were tested positive for H7 and H5 and suggest low or no prevalence of these viruses in poultry. The latter was confirmed by the absence of H5 and H7 AIVs in oropharyngeal swabs of poultry in the current study. Similar has been reported in various studies previously.^17^, ^30^, ^41^, ^42^ Although H5N1 has persistently circulated in poultry in many other countries, especially in Asia, human infection has rarely been reported.^24^, ^43^, ^44^, ^45^, ^46^ H5 viruses have shown strong host specificity for infection, which could be the reason for the low infection rate in poultry exposed people even in countries enzootic for the disease.^17^, ^47^

Overall, 15.5% H9 seroprevalence was found in occupationally exposed butchers providing serological evidence of human infection with antigenically similar viruses.^48^ It was significantly higher (15.5% vs 6%) than controls subject (*P* < .05) and was strongly associated with seroprevalence (89%) in live birds in stall (*P* < .001) suggesting poultry‐to‐human transmission of H9 AIV in these LPRSs instead of human‐to‐human contact.^21^ These results also provide confidence in the cutoff value (titer ≥ 40); we used to score seropositivity. Chakwal has a high density of commercial poultry,^49^ and poultry handlers from this area are expected to have the highest level of exposure to AIV infected birds during slaughtering and handling.

Previous studies in Pakistan^15^ and other countries^17^, ^30^, ^34^, ^43^, ^44^ estimated that H9 seroprevalence ranged from 0.7% to 50% in poultry workers. Very few virologically conferred H9N2 human cases have been reported to the World Health Organization. This disparity in serological versus virological measures of infection indicate the existence of considerable asymptomatic or mild H9N2 infections among exposed humans. This also is consistent with the relatively mild nature of those H9N2 cases that have been reported. The mild upper respiratory tract illness associated with H9N2 infection makes it indistinguishable clinically from influenza‐like illnesses caused by seasonal influenza viruses (H1N1 and H3N2) contributing to its underestimated incidence.^24^ The differences in reported seroprevalence across published studies could be at least partially explained by differences between serological assays with different sensitivity/specificity and high inter‐study variability. H9N2 viruses have been known to infect humans since 1998,^24^, ^44^, ^46^ and its position as a candidate to cause an influenza pandemic has been suggested.^18^, ^50^, ^51^

Various determinants for human infection with AIV have been documented.^46^, ^52^ In current study, presence of another LPRS nearby and number of cages in the stall were strongly associated with seroprevalence of H9 (*P* < .05). Both these factors are linked to poultry density, that is, a nearby poultry stall will increase the poultry density; similarly, the number of cages also reflects bird density. An increased number and density of live poultry may increase virus circulation and increases the risk of infection, subsequently increasing the probability of virus dissemination and genetic reassortment.^53^, ^54^, ^55^, ^56^ Other studies have reported risk factors such as handling dead birds, butchering, area of farm, swimming or bathing in household ponds, having sick or dead poultry, age, vaccination of poultry, and exposure to ducks.^15^, ^45^, ^46^ In experimental studies, influenza virus has been recovered from air samples during slaughter of asymptomatic H5N1 HPAIV infected chickens and ducks, and exposure of chickens and ferrets within the same air space as the slaughter process resulted in airborne transmission and infection indicating a plausible method for transmission of AIV in LPMS to poultry and humans, respectively.^57^, ^58^ For reasons not immediately clear, these risk factors were not significant for H9N2 infection in our study.

Since 1998, various G1‐lineage H9N2 viruses have been reported in Pakistan and are continuously evolving through reassortment with H5N1 and H7N3 HPAIVs, subsequently generating a new H9N2 genotype. All reported H9N2 viruses collected during 2009, 2010, 2012, and 2015 in Pakistan were reassortants between the G1 lineage and the H7N3 HPAIV that circulated in Pakistan and contained several mammalian host‐specific markers.^18^, ^26^ Since 2015, no further genetic sequence has been reported. In this study, 3 out of 11 pooled swab samples from broiler birds were positive for H9, which showed that H9N2 virus is still circulating in poultry in Pakistan. H9N2 reassortants viruses have been linked to the emergence of numerous AIVs (H5N1, H7N9, H10N8) donating internal genes, raising public health concern globally.^59^, ^60^ Concurrent circulation of H9N2 reassortants in LPRSs and evidence of human exposure to these circulating viruses represents a major risk for emergence of new pandemic in both occupationally exposed poultry handlers and the general population in Pakistan. Due to the presence of mammalian host‐specific markers in these H9 viruses, they pose a serious threat to public health in Pakistan.

In current study, small sample size has put limitation on the generalization of estimates produced. Due to small number of H9‐seropositive persons (n = 25), the power of study may not be efficient to detect significant differences for some potential risk factors. Furthermore, the current study was a cross‐sectional survey; these study designs are not suited to estimate disease incidence, natural history of disease, or the rate of secondary human‐to‐human infection. Cross‐sectional studies are also relatively weaker in establishing causality of risk factors than with an analytic design, such as with a cohort study.^61^ Well‐designed prospective epidemiological studies with follow‐up of human and their birds will be better suited to answer such questions.^46^ The authors acknowledge that including control human sera from repository of a private laboratory in Chakwal, which were not collected in the same time frame in which the butchers’ sera were collected, might have introduced bias in our results.

## 5 CONCLUSIONS

In summary, we have documented seroprevalence of H9 in butchers in LPRSs of Chakwal. We also established evidence of co‐circulation of H9 virus in poultry in the same LPRS, which poses a continued threat for emergence of novel genotypes of AIVs through intra‐ and inter‐subtypic reassortment. Governmental interventions to mitigate the prevalence of AIVs in these markets may reduce the risk for emergence of novel viruses. Continued active surveillance and genetic characterization of H9N2 are highly recommended to detect any ongoing public health risk.^33^ Studies using one‐health strategy, combined with clinical and virological surveillance in target population, would be needed to document any cross‐species transmission of novel avian influenza viruses.^62^
