# Supplementary material for: Avian influenza at animal‐human interface: One‐health challenge in live poultry retail stalls of Chakwal, Pakistan
Source: Influenza Other Respir Viruses. 2020 Feb 7;14(3):257–65. doi: 10.1111/irv.12718 (PMC7182597; doi:10.1111/irv.12718)
Supplement: Supplementary file 1 [file IRV-14-257-s001.docx]

**RISK FACTORS OF AVIAN INFLUENZA (H5, H7, H9) AMONG BUTCHERS IN DISTRICT CHAKWAL**

This section must be filled in before completing the questionnaire

| **ID#** |  |  |  |
| --- | --- | --- | --- |

| Date: | District ID: |
| --- | --- |
| Area Name: | Market Name: |
| Latitude: | Longitude: |
| Name of Interviewer: | |
| Name of Respondent: | Telephone no: |
|  | |
| Consent to participate (Written) Yes No | |

1-How old are you?........................................................_______________________ Years

2. Education level .........................uneducated Primary Secondary and above

3-Do you smoke? ......................................................... Yes No (if no, skip to 4)

4-How many sticks a day do you smoke? ..................... _______________

5-How many years have you smoked? ......................... _______________

6-Do you have any chronic disease history?........................................... Yes No

7. If yes to above question, which disease you have............................................................

Asthma Diabetes Chronic heart disease chronic lung disease

Chronic liver disease chronic kidney disease Cancer

8-How many days of a week, this stall remains open?

Seven Six Five Four Three Two One

9-How many cages do you have in your stall?............................... Less than 5 more than 5

10-On average, approximately how many birds are sold per day?.................. < 100 >100

11- Do you sell birds other than broiler..............................................................Yes No

12-Do you add newly arriving birds to cages that already contain birds? Yes No

13-How often do you see wild birds around your stall? .............. Usually Rarely

14-How often do you see rodents (rats and mice) in your stall?

………………………………………………………………. Usually Rarely

15-Do you keep birds at home? ...........................................................Yes No

16-Do stray dogs have access to stall? .............................................. Yes No

17-Do stray cats have access to stall? ................................................ Yes No

18- Do you have any other stall nearby? ............................................ Yes No

19-Do you prepare raw poultry and other foods using different knifes? Yes No

20-Is there any hand washing facility in the market (interviewer to observe and record)

………………………………………………………………………… Yes No

21-How you wash gizzard of slaughtered poultry? ......................................................

………………….…………. Dip in bucket of water separately under tap

22-Do you touch your face/food after touching carcasses of poultry? ……. Yes No

23-Do you wash instrument for slaughtering one birds after slaughtering other birds?

…………………………………………………………………………........Yes No

24-Do you clean cutting boards after preparing raw poultry? ...................... Yes No

25-Do you wash your hands before and after slaughtering each bird? ……... Yes No

26-Do you wearing mask during poultry slaughtering and handling?........... Yes No

27- Do you wearing gloves during poultry slaughtering and handling?......... Yes No

28- Do you wearing approns during poultry slaughtering and handling?....... Yes No

29- Do you wearing protective boots during poultry slaughtering and handling?................

………………………………………………………………………………..Yes No

30-Do you cover your nose and mouth with a handkerchief when sneeze?.... Yes No

31-After slaughtering do you put carcasse on ground/in drum?..................... Yes No

32-Feathers scattered in the air/on the ground?............................................... Yes No

33-Do you wash your stall daily including table,floors,walls etc?……………Yes No

Are you interested in follow up sample collection? .................................. Yes No
